# Molecular basis and homeostatic regulation of Zinc taste

**DOI:** 10.1007/s13238-021-00845-8

**Published:** 2021-04-23

**Authors:** Rui Luo, Yuxiang Zhang, Yinjun Jia, Yan Zhang, Zongyang Li, Jieqing Zhao, Ting Liu, Wei Zhang

**Affiliations:** 1 School of Life Sciences, IDG/McGovern Institute for Brain Research, Tsinghua University, Beijing, 100084 China; 2 Tsinghua-Peking Center for Life Sciences, Beijing, 100084 China

**Dear Editor,**

Among all the essential trace metals, zinc is of particular importance for its huge panoply of biological functions. As a pivotal element for hundreds of enzymes, either scarcity or accumulation of body zinc can lead to severe disorders (Maret, 2017). Therefore, body zinc must be tightly regulated in order to keep a precise amount that is sufficient for cells’ survival but does not cause toxicity. As diet is the major source of trace elements including zinc, animals must adjust their feeding amount and preference to avoid scarcity or accumulation. However, how animal utilize their gustatory organs to detect zinc in food and whether the taste is regulated with body zinc level are unknown. Metals often cause the sense of taste termed as metallic taste. While sodium of certain concentration range delivers hedonic salty taste, salt solutions of most metals taste aversive. It was reported that zinc, copper and other heavy metal ions trigger an unpleasant sense of astringent/drying, sour or bitter. Although taste receptor cells were found essential for the detection of some divalent ions, the molecular mechanisms for this detection are largely elusive. Recently, the receptors for calcium were unambiguously identified in fruit fly (Lee et al., 2018). However, a full repertoire of receptors for sensing divalent ions is still lacking.

To ask whether flies are capable of tasting zinc in food, we adopt a binary choice approach to test flies’ taste preference to zinc-containing food (Tanimura et al., 1982; Lee et al., 2018). In a 48-well plate, half wells were supplied with 5 mmol/L sucrose solution in agarose and the other halves with 5 mmol/L sucrose solution plus zinc gluconate at different concentrations in an alternative manner. Each half was colored with food dyes to either red or blue (Fig. 1A). Under such an assay, flies avoided food containing zinc in a dose-dependent manner, indicating zinc in food suppressed feeding (Fig. 1A).

**Figure 1:**
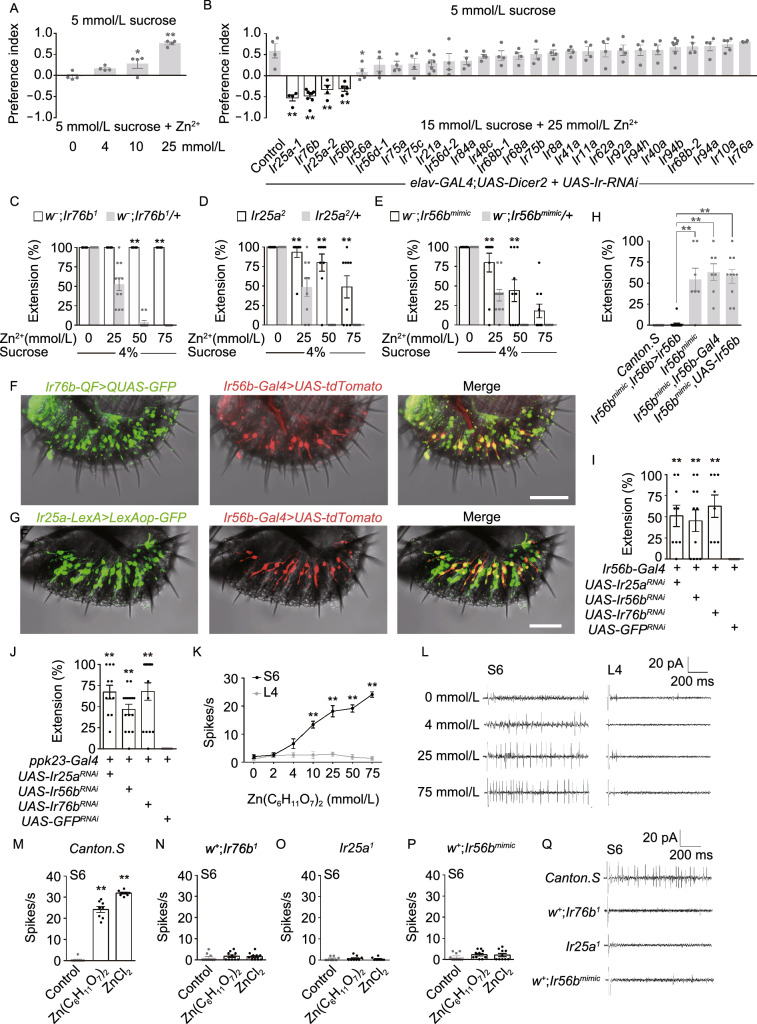
**Three ionotropic receptors mediate fly’s distaste to zinc**. (A) Two-way choice taste assay for a group of 50–80 wildtype files (*Canton. S*) per assay. Preference index of files between 5 mmol/L sucrose alone and the same concentration of sucrose with different concentrations of zinc gluconate. One-way ANOVA followed by *post hoc t* test with Bonferronicorrection; *n* = 4. (B) Two-way choice assays for screening candidate IR family receptors for aversive taste to zinc gluconate. GFP-RNAi was used as a control. One-way ANOVA followed by *post hoc t* test with Bonferroni correction; *n* = 4–8. (C–E) Dose-dependent taste aversion of Ir76b, Ir25a and Ir56b mutants to indicated concentration of zinc gluconate. Two-way ANOVA followed by *post hoc t* test with Bonferroni correction; *n* = 6–11. (F) Expression of *Ir76b-QF* (*GFP*) and *Ir56b-Gal4* (*tdTomato*) on the labellum. (Scale bars: 50 μm.) (G) Expression of *Ir25a-LexA* (*GFP*) and *Ir56b-Gal4* (*tdTomato*) on the labellum. (Scale bars: 50 μm.) (H) PER to 50mmol/L zinc gluconate in 4% sucrose of Ir56b mutant flies and genetic rescue of Ir56b in zinc GRNs. One-way ANOVA followed by *post hoc t* test with Bonferroni correction; *n* = 7–16. (I and J) PER of files with genetic knockdown of Ir25a, r56b or Ir76b to 50 mmol/L zinc gluconate supplement of 4% sucrose in Ir56b neurons (I) and ppk23 neurons (J), respectively. One-way ANOVA followed by *post hoc t* test with Bonferroni correction; *n* = 7–15. (K) Tip recordings from the indicated sensilla in response to different concentration of zinc gluconate. Two-way ANOVA followed by *post hoc t* test with Bonferroni correction; *n* = 5–8. (L) The representative traces of spikes from the indicated sensilla in response to different concentration of zinc gluconate. (M–P) Comparing WT with Ir76b, Ir25a or Ir56b mutant diminished the zinc-triggered spikes to zinc salt. One-way ANOVA followed by *post hoc t* test with Bonferroni correction; *n* = 8–10. (Q) Representative traces of spikes from indicated genotype of flies to 75 mmol/L zinc gluconate. For all comparisons performed in this figure, *P < 0.05, **P < 0.01

To explore whether the zinc repulsion was mediated by gustatory organs, we used the proboscis extension response (PER) assay to assess flies’ taste sensitivity to zinc by directly stimulating the flies’ labellum. Flies showed strong PER to 4% sucrose (ca. 117 mmol/L) (Figs. S1B and S5I). However, when zinc gluconate was added to the solution, the response was inhibited in a concentration-dependent manner (Fig. S1B); gluconate was not responsible for the aversive taste as magnesium gluconate did not affect PER to sucrose at the concentration range tested (Fig. S1B). Moreover, ZnCl_2_ caused a similar taste repulsion as zinc gluconate did (Fig. S1B). Given that Cl^−^ was tasteless to flies (Zhang et al., 2013; Jaeger et al., 2018; Lee et al., 2018), the aversive taste of zinc salt was from zinc rather than the acidic groups. Additionally, ZnSO_4_, a chemical that is used of zinc deficiency diagnosis for human (Gruner and Arthur, 2012), elicited a comparable aversion to flies as other zinc salts did (Fig. S1B). Together, zinc delivered an aversive gustatory signal via taste organs on the labellum.

In flies, the activation of bitter gustatory receptor neurons (GRNs) suppresses PER and feeding (Fig. S5J). To test whether zinc conveys the aversive taste via bitter GRs, we tested the PER response of the mutants of two bitter GRs: Gr32a and Gr33a. The mutants for the two GRs showed normal repulsion to zinc (Fig. S1D); further, when another bitter GR (Gr66a) was knocked down in Gr66a-expressing GRNs with dsRNA, the taste sensitivity to zinc was not affected (Fig. S1C). We also tested several other bitter GRs (Gr47a, Gr89a, Gr93a, Gr10a, Gr8a, Gr28b and Gr98b). None of them appeared to play a role in zinc taste (Fig. S1D). As these GRs mediated the aversive taste to most known bitter compounds (Chen and Dahanukar, 2020), the results argue that zinc taste was mediated with other receptors rather than the bitter GRs. We next examined several chemo-TRPs that were reported to detect aversive components in food. Painless, dTPRA1 and dTRPM, a channel that was found permeable to zinc, were all dispensable for zinc sensation (Fig. S1D).

What’s the molecular basis for zinc taste in flies? Previous studies have revealed that members of the ionotropic receptor (IR) family are essential for flies to taste salts (Zhang et al., 2013; Jaeger et al., 2018; Lee et al., 2018). We thus turned our attention to IRs. By two-way choice assays using 5 mmol/L sucrose alone versus 15 mmol/L sucrose plus zinc gluconate (Fig. S1A), we were able to introduce a bias preference to the flies so that the zinc avoidance became more evident. Without zinc, the flies preferred the sweeter food at 15 mmol/L sucrose, resulting in a negative preference index (PI) (Fig. 1B). As the zinc concentration increased, the propensity of choosing the less sweet food grew, demonstrating that zinc triggers aversive taste to overcome the preference for sweeter food. At a concentration of 25 mmol/L zinc, the PI for sweet food was 0.31, indicating that this zinc concentration was sufficient for the flies to unambiguously distinguish the zinc-containing food but was not saturated so that it would cause false-negative effect during the screening.

With this assay, we examined 27 RNAi lines affecting 24 IR genes and found that knocking down Ir25a, Ir76b or Ir56b pan-neuronally strongly impaired the taste avoidance to zinc (Fig. 1B). In contrast, Ir62a, a receptor found to mediate the aversive taste to calcium, was dispensable for the detection of zinc, indicating that Ir56b and Ir62a each plays specific role in sensing zinc and calcium salts, respectively. To further validate the results, we tested the roles of Ir25a, Ir76b and Ir56b in zinc detection with PER assay. Knocking down all these three IRs pan-neuronally resulted in a remarkably reduced aversive response to zinc (Fig. S1E). Additionally, mutant flies for Ir25a, Ir76b or Ir56b exhibited a defective taste sensitivity to zinc (Figs. 1C–E, S1F and S1G). We confirmed the *Ir56b*^*mimic*^ allele was a loss-of-function mutant of the Ir56b gene (Fig. S1H). Given that the *Ir76b*^*1*^ and *Ir56b*^*mimic*^ were in *w*^*1118*^ background, we back-crossed the mutant strains with Canton S. and tested their sensitivity to zinc. The mutants and wildtype flies with the same background were grouped together for comparison. The similar defect was observed (Fig. S1F and S1G). While Ir76b and Ir25a were found to be important for sensing other salts (Chen and Dahanukar, 2020), Ir56b appeared more narrowly tuned, as its mutant showed normal taste response to sodium and calcium salts (Fig. S1L and S1M). Taken together, these findings revealed that the three IRs were functionally required for the aversive taste response to zinc.

Ir25a and Ir76b were found broadly expressed in the GRNs under the taste sensilla on the labellum (Lee et al., 2018; Sanchez-Alcaniz et al., 2018). In contrast, Ir56b only expresses in S- and L-type taste sensilla (Koh et al., 2014). By double-labeling experiments, we have confirmed the previous findings that Ir25a and Ir76b were co-expressed in the majority of the GRNs (Fig. S1I) while Ir56b was expressed in a sub-population of GRNs that are labelled with both Ir25a and Ir76b (Fig. 1F and 1G), suggesting the GRNs with overlapping expression of the three IRs were essential for zinc taste. To test this notion, we carried out GRN-specific mutual knockdown experiments. Reducing the mRNA levels of Ir25a, Ir76b and Ir56b in any of the three GRN classes dampened the flies’ taste sensitivity to zinc (Figs. 1I, S1J and S1K). Notably, knocking down Ir56b in any of the three GRN classes resulted in a less severe defect in zinc taste (Figs. 1I, S1J and S1K). This result echoed the weaker phenotype for zinc taste defect in Ir56b mutant and a smaller number of Ir56b expressing GRNs (Fig. 1F and 1G). Importantly, the zinc taste defect of *Ir56b* mutant can be rescued by expressing Ir56b with Ir56b-Gal4, further validating the specificity of Ir56b in sensing zinc (Fig. 1H). In this way, we identified a class of GRNs (namely zinc GRNs) that expressed Ir25a, Ir76b and Ir56b to detect zinc and mediated the aversive taste response.

This finding reminded us that a similar strategy was used in flies to sense calcium: Ir25a, Ir76b and Ir62a function in ppk23-expressing neurons to mediate the aversive taste to calcium (Lee et al., 2018). Ppk23 was also found essential for detecting sodium in the food (Jaeger et al., 2018). It was thus proposed that ppk23 marks a class of GRNs that are tuned to salts. To test whether a similar mechanism applies to zinc, we blocked the activity of the ppk23-expressing GRNs with an inward-rectifying potassium channel Kir2.1. This resulted in a greatly reduced aversion to zinc in a dose-dependent manner (Fig. S2A); Furthermore, knocking down Ir25a, Ir76b or Ir56b in ppk23 neurons phenocopied the defect for zinc sensation observed in the mutants of the three IRs (Figs. 1J, 1I, and S1J and S1K).

Double labelling between IRs and ppk23 revealed that ppk23 GRNs were a sub-class of Ir76b and Ir25a GRNs (Fig. S2E and S2F) while Ir56b marked a sub-population of ppk23 GRNs (Fig. S2G). Interestingly, the ppk23 gene itself appeared dispensable for zinc taste as its mutant alleles showed normal aversion to zinc-containing food, similar to its role observed in calcium taste (Lee et al., 2018) (Fig. S2B). The zinc sensing neurons appeared separate from those mediating aversive bitter taste. Bitter GRNs (labelled with Gr66a) were not required for zinc taste, as silencing them had no impact on the PER to zinc-containing food (Fig. S2C), nor did knocking down any of the three IRs in those neurons (Fig. S2D). Double labelling validated that Ir56b and Gr66a were not express in the same group of GRNs (Fig. S2H). The results above demonstrated that a subset of ppk23-expressing salt GRNs on the labellum mediate the aversive taste response to zinc.

GRNs underneath the taste sensilla on the labellum fire action potentials upon gustatory stimulation and the spikes can be monitored with the tip recording method. Among the taste sensilla, S-type sensilla respond to aversive bitter stimuli while L-type sensilla are tuned to sweet tastants . As Ir56b is expressed in S6 sensilla, we carried out recording on S6. The GRNs in S6 exhibited a robust spike firing in a concentration dependent manner upon touching the zinc solution (Fig. 1K and 1L). This response was dependent of Ir25a, Ir76b and Ir56b as mutation of any of the three IRs almost completely diminished the zinc-triggered spikes (Fig. 1M–Q). We have performed tip recordings on S6 sensilla in flies with Ir25a, Ir56b and Ir76b knocked down in taste neurons with the ppk23-Gal4 and recorded the spike responses to zinc and denatonium benzoate (DEN), a bitter substance. The results further demonstrated that the three IRs are functionally important for the taste neurons’ normal response to zinc but not to DEN (Fig. S3A–E). ZnCl_2_ and zinc gluconate elicited an equally robust spike response (Fig. 1M), suggesting that the activity was specific to zinc. In contrast, GRNs in the L-type bristles did not exhibit an evident response to zinc but responded strongly to sugar (Figs. 1K, 1L, S3F and S3G). Moreover, the sugar induced action potentials of sweet GRNs were not suppressed by adding zinc to the solution (Fig. S3F and S3G), differing it from calcium and some bitter substances (Lee et al., 2018). Taken together, Ir25a, Ir76b and Ir56b act together to confer zinc sensitivity to a subset of ppk23-expessing salt GRNs under S-type sensilla to detect zinc in food.

So far, we have demonstrated that there IRs function in a subset of gustatory receptor neurons to detect zinc in the food and mediate the aversive behavior. IRs are found essential in sensing several other cations. Ir76b is required for sensing sodium, one of most abundant cations in the fly diet (Zhang et al., 2013); Ir76b, Ir25a and Ir62b were reported to convey aversive taste from calcium-containing food (Lee et al., 2018). Moreover, IRs are receptors for tastants such as fatty acid and organic acid (Chen and Dahanukar, 2020). As Ir76b and Ir25a are broadly tuned to many tastants and they are ubiquitously expressed in most GRNs on the labellum, it’s conceivable that they function as co-receptors (Jaeger et al., 2018; Sanchez-Alcaniz et al., 2018). In contrast, several other IRs exhibit high selectivity to only certain tastant (Lee et al., 2018; Sanchez-Alcaniz et al., 2018). Nevertheless, we found that the three IRs Ir25a, Ir76b and Ir56b may not be sufficient to convey zinc sensitivity by themselves, as heterologous expression of Ir56b with Ir76b-Gal4 did not convey zinc sensitivity to the L-type sensillia, though Ir76b and Ir25a were found expressed in those sensilla (Fig. S3H and S3I). This result argues that the three IRs Ir25a, Ir76b and Ir56b were not sufficient to convey zinc sensitivity to heterologous taste neurons. An essential component of the receptor complex or a unique structure may render the naive Ir25a/Ir76b/Ir56b positive neurons the ability to sense zinc.

Excessive zinc intake causes lethality to fish and fly larvae (Rehwoldt et al., 1971; Wang et al., 2009). To test whether this holds true for adult flies, we tested the survival rate of flies maintained on 100 mmol/L fructose (Lee et al., 2018) or on fructose supplied with different concentrations of zinc. Flies’ viability was not dramatically reduced for up to a week fed on fructose (Fig. S4A). In contrast, zinc addition to the food induced early lethality at the concentration of 2 mmol/L (Fig. S4A) and this effect plateaued after 10 mmol/L.

It’s plausible that the aversive taste to high zinc in food provides a protective mechanism for the flies to avoid toxic food. To exclude the possibility that the stress induced by prolonged exposure to an aversive food source may lead to lethality, we adopted a binary food-choice survival assay that allowed the flies to choose freely between 100 mmol/L fructose alone and 200 mmol/L fructose containing 50 mmol/L Zinc. Wild type control flies succeeded in avoiding the zinc containing food while the mutants for Ir76b or Ir56b failed to do so, resulting in a significant reduction of viability (Fig. S4B). These results support the notion that high zinc upon feeding induces lethality and the zinc IRs endow the flies with the ability to avoid such a toxicity.

Up to this point, we have shown that flies can taste zinc in the food and avoid high concentration zinc. Given that low body zinc level causes zinc taste defects in human and other mammals (Gruner and Arthur, 2012), we asked whether flies’ gustatory sensitivity to zinc is also tuned by body zinc. To explore this idea, we fed the flies with food containing the zinc-specific chelator N,N,N’N’-tetrakis-(2-pyridylmethyl) ethylenediamine (TPEN) that lowers the body zinc level than flies reared on normal food (NF) (Wang et al., 2009; Tejeda-Guzman et al., 2018). We first confirmed that feeding flies with TPEN did not affect their overall food consumption (Fig. S5A). By PER assay, we found the zinc depleted flies but not those fed with ethanol as a solvent control showed a reduced aversion to zinc-containing food (Fig. 2A). The taste sensitivity to zinc in the zinc-depleted flies was restored after they were reared in normal food for two days (Fig. 2B). Notably, feeding flies with food with 5 mmol/L zinc supplement did not shift their zinc sensitivity to a higher level (Fig. S5B), indicating that the flies are able to maintain the normal zinc sensitivity after the ingestion of excessive zinc. We also performed tip recording on S6 bristle under the zinc depletion condition and found that body zinc deficiency from TPEN feeding caused a reduction of zinc-triggered spike firing in the zinc GRNs (Fig. 2C and 2D).

**Figure 2:**
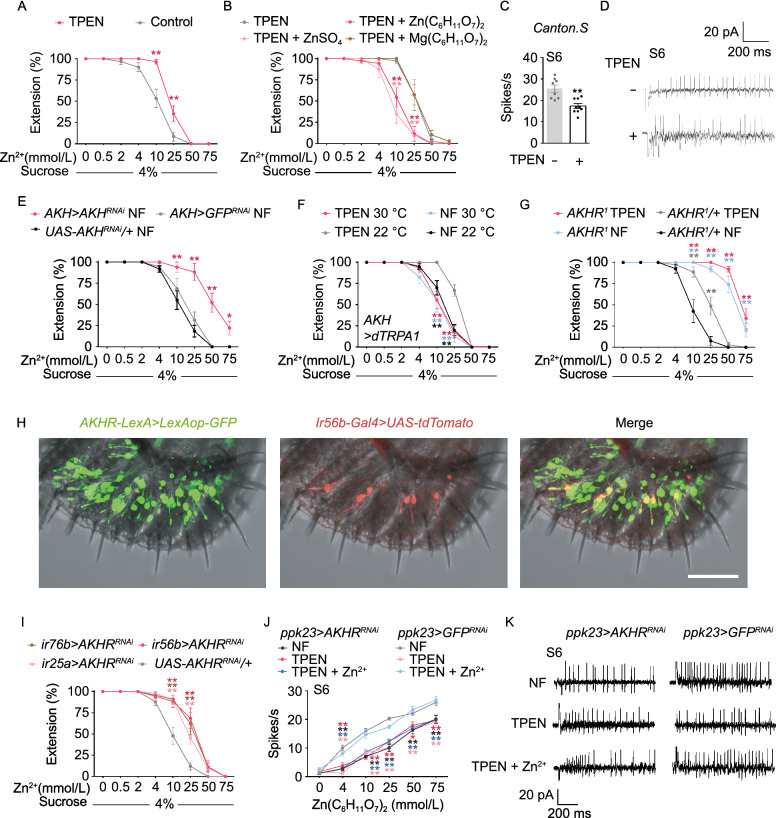
**Zinc taste is regulated by diet via AKH signaling**. (A) PER of WT flies with zinc-deficiency diet or control diet to different concentration of zinc gluconate supplement of 4% sucrose. Two-way ANOVA followed by *post hoc t* test with Bonferroni correction; *n* = 9–17. (B) After 4 day of zinc depletion, PER of WT flies was tested when fed with zinc-supplement or magnesium-supplement diet for two days. Two-way ANOVA followed by *post hoc t* test with Bonferroni correction; *n* = 7–11. (C) Tip recordings in response to 75 mmol/L zinc gluconate of S6 sensilla in flies with zinc-depletion or with normal food diet. Nonparametric Mann-Whitney test; *n* = 8–9. (D) Representative traces of spikes from S6 sensilla of files with zinc-depletion or normal food diet in response to 75 mmol/L zinc gluconate. (E) PER of flies in normal food (NF) with genetic knockdown of AKH in AKH producing neurons. Two-way ANOVA followed by *post hoc t* test with Bonferroni correction; *n* = 7–12. (F) PER of flies with heat activation of AKH producing neurons. Two-way ANOVA followed by *post hoc t* test with Bonferroni correction; *n* = 7–11. (G) PER of control flies and AKHR^1^ mutant on zinc-deficient or normal food diet. Two-way ANOVA followed by *post hoc t* test with Bonferroni correction; *n* = 6–11. (H) Expression of *AKHR-LexA* (*GFP*) and *Ir56b-Gal4* (*tdTomato*) on the labellum. (Scale bars: 50 μm.) (I) PER of flies with genetic knockdown of AKHR in Ir76b, Ir25a or Ir56b neurons. Two-way ANOVA followed by *post hoc t* test with Bonferroni correction; *n* = 7–18. (J) Tip recordings in response to indicated concentrations of zinc gluconate of S6 sensilla in flies with genetic knockdown of AKHR or control in zinc-depletion, normal food diet or zinc-supplement diet after zinc depletion. Two-way ANOVA followed by *post hoc t* test with Bonferroni correction; *n* = 6–8. (K) Representative traces of spikes from S6 sensilla of files with indicated diet in response to 75 mmol/L zinc gluconate. For all comparisons performed in this figure, *P < 0.05, **P < 0.01

Body zinc homeostasis of flies is maintained by a cohort of zinc transporter proteins. Among them, ZnT1 was found to play an essential role for absorbing zinc from food by escorting zinc from the enterocytes to hemolymph (Wang et al., 2009). Mutant of ZnT1 resulted in a reduced body zinc level (Wang et al., 2009). We found taste sensitivity to zinc of this mutant was impaired than wildtype flies when reared in normal food (Fig. S5C). These results indicate that body zinc level that is controlled with diet or nutrition absorption processes can regulated zinc taste sensitivity reversibly. Further evidence came from the observation that *w*^*1118*^ flies were less sensitive to zinc compare to *Canton S*. It was known that the *white* gene is required for normal body zinc level (Tejeda-Guzman et al., 2018). One cope of the *white* gene or the *mini-white* transgene was sufficient to shift the sensitivity of *w*^*1118*^ to Canton S. level, while multiple *mini-white* transgenes did not further enhance the sensitivity (Fig. S5K). Additionally, adding zinc to the fly food ameliorated the defective zinc acuity of *w*^*1118*^ while feeding *Canton S* flies with TPEN reduced their zinc sensitivity (Figs. 2A and S5D).

What is the mechanism by which body zinc affects zinc taste? Studies *in vitro* and in animal models have shown that zinc plays important roles in the secretion of insulin and its key counterregulatory hormone glucagon (Maret, 2017). To ask whether the glucagon-like AKH pathway may participate in the zinc taste regulation, we first blocked the AKH producing neurosecretory neurons in the corpora cardiac (CC) with Kir2.1. Silencing these neurons greatly dampened the flies taste sensitivity to zinc (Fig. S5E). In parallel, knocking down AKH in the corpora cardiac neurons resulted in a similar phenotype (Fig. 2E). This zinc dysgeusia caused by the blockage of AKH secretion was not further exacerbated by dietary zinc depletion (Fig. S5E), implicating that AKH secretion is a downstream of body zinc level change. Indeed, activation of the corpora cardiac neurons with the warm-activated cation channel TRPA1 reversed the reduction of zinc taste sensitivity caused by zinc depletion but did not further elevate it when the flies were fed with normal food (Fig. 2F).

We next asked what is the target of the AKH peptide –in the regulation of zinc taste. AKHR, the receptor of AKH, is broadly expressed in neurons and other tissues to regulate metabolism, feeding and water intake, etc. (Inagaki et al., 2014). To begin discerning the role of AKHR in zinc taste regulation, we tested the PER to zinc-containing sugar food of an *Akhr* mutant. The flies showed a reduced repulsion to zinc (Fig. 2G), comparable to the flies in which the AKH secretion was blocked (Fig. S5E), suggesting AKHR is essential for the zinc taste regulation by body zinc level. To investigate whether this effect was mediated with neuronal AKHR, we knocked down AKHR expression with the pan-neuronal driver Elav-Gal4. Again, the flies lost the ability to adjust their zinc taste sensitivity to different zinc diet (Fig. S5F).

But does AKHR function on the GRNs or their downstream neural circuits? To differentiate between the two possibilities, we used ppk23-Gal4 to knock down AKHR in the salt GRNs and observed a similar defect of zinc taste regulation compared to the pan-neuronal knockdown experiments (Fig. S5F). Furthermore, knocking-down AKHR in Ir76b, Ir25a or Ir56b GRNs was sufficient to diminish the regulatory effect of zinc diet on zinc taste (Fig. 2I). To further validate that AKHR functions in GRNs, we co-labelled the zinc GRNs and AKHR-expressing neurons. We found AKHR is expressed in the majority of the labellar zinc GRNs (Figs. 2H, S5G and S5H). We also performed tip recordings on taste sensilla of flies with AKHR knockdown in the taste neurons. The sensitivity to zinc was reduced at all zinc concentrations tested, albeit maintaining the dose-dependent manner (Fig. 2J and 2K).

The glucagon-like AKH-AKHR signaling has been found to regulate taste sensitivity under different feeding conditions in both flies and rodents (Inagaki et al., 2014). We here showed that AKH directly targets on the gustatory receptors neurons and tunes their activity. This mechanism allows fast and specific regulation of taste perception for the nutrients. Although our results demonstrate that body zinc acts upstream of the AKH-AKHR pathway, it’s still an open question whether zinc directly stimulates AKH secretion from CC. The glucagon pathway may play a similar role in mammals, a notion supported with the fact that a variety of neuropeptides are expressed in the taste receptor cells on the tongue, including the glucagon receptors. Nevertheless, the homeostasis of body zinc must be regulated with multifaced mechanisms. A recent finding demonstrated that a zinc-gated chloride channel in the fly gut mediates the dietary preference for zinc (Redhai et al., 2020). It’s remaining to be explored whether these regulation machineries are converged to the same pathway.

It was reported that zinc deficiency can cause general neurosensory disorder including loss of taste to many tastants (Gruner and Arthur, 2012). We observed a similar defect in the flies with zinc depletion. In the test for sweet taste, the flies with zinc deficiency showed a lower PER to sugar food (Fig. S5I). In contrast, in the PER test to 4% sucrose containing DEN, the aversive taste was also weakened in the zinc depleted flies (Fig. S5J). Intriguingly, only the regulation of bitter taste appeared to be mediated with the AKHR signaling, as knocking down AKHR pan-neuronally with Elav-Gal4 had no impact on the regulation of sweet taste by zinc depletion (Fig. S5I) but blocked the reduction of aversive taste to bitter substance caused by zinc deficiency (Fig. S5J). We next asked with the AKH-AKHR axis may also contribute to the regulation of other salt taste. We tested the flies’s taste sensitivity to calcium and sodium under different feeding conditions. It appeared that the PER responses to the two metal ions were not changed under normal food feeding or zinc-depletion condition (Fig. S5L and S5M), arguing that the functions of the AKH/AKHR axis in regulating taste ion sensitivity is zinc selective.

In healthy human, zinc causes strong aversive oral sensation. This taste becomes less intense when the people are of low body zinc (Gruner and Arthur, 2012). Here we find the similar mechanism holds true for insects. Flies utilize dedicated receptors and gustatory neurons to detect zinc in the food and to suppress ingestion. As constantly feeding on zinc-containing food causes lethality, this distaste to zinc must be critical for flies in the natural environments to avoid the food sources with high zinc concentration. Indeed, zinc level in some fruit and leaves are high enough (Mansour, 2019) to elicit aversive taste to flies. Notably, zinc does not become attractive even at very low concentration, indicating that in the natural zinc is usually abundant and flies can readily get enough zinc from regular feeding. Conversely, a group of central neuroendocrine cells that secrete AKH monitor low body zinc to temper zinc taste aversion therefore promotes zinc ingestion and maintains zinc homeostasis.

## Supplementary Information

Below is the link to the electronic supplementary material.

Supplementary material 1 (PDF 57568 kb)
