# Supplementary material for: Molecular basis and homeostatic regulation of Zinc taste
Source: Protein Cell. 2021 Apr 23;13(6):462–9. doi: 10.1007/s13238-021-00845-8 (PMC9095774; doi:10.1007/s13238-021-00845-8)
Supplement: Supplementary file 1 — Supplementary material 1 (PDF 57568 kb) [file 13238_2021_845_MOESM1_ESM.pdf]

Materials and Methods

Animals. Files were raised on standard cornmeal medium (10 g agar, 7.25 g sucrose, 30 g glucose, 24.5 g yeast, 50 g corn meal, 17.5 ml methyl 4-hydroxybenzoate, 4ml propionic acid in one 1L) with a 12h/12h light/dark cycle at 25 ℃ in 60% humidity. For behavioral and tip-recording assays, the cross for RNA interference for *AKHR* were maintained at 27℃ for 9 days before experiment (Rajan et al., 2017). For the group of flies with zinc-deficiency diet or external zinc-supplement diet, 100 μM TPEN (SIGMA, 16858-02-9) or 5 mM zinc gluconate was add to standard cornmeal medium respectively, which was reported not lethal to flies (Richards et al., 2015) and the foods source would substitute to files 4 days before experiment. TPEN was dissolved in ethanol and added to the standard cornmeal medium to reach the final concentration. 0.1% ethanol was added to the food as control. To exclude the contribution of transgenic *white* gene to zinc sensitivity, we back-crossed the white-eye mutant strains with *Canton S* and obtained red-eye mutant strains.

Flies. *Canton.S* (BL64349) were used as wild-type control. The following transgenic lines were used: w^{1118} (BL5905), $Gr33a^{1}$ (BL31427), $Gr32a^{KO}$, $Gr8a^{1}$ (BL40976), $Gr10a^{mimic}$ (BL29947), $Gr28b^{mimic}$ (BL24190), $Gr47a^{1}$ (BL65843), $Gr93a^{f01688}$ (BL18458), $pain^{KO}$, $TrpA1^{ins}$, $Ir76b^{1}$ (BL51309), $Ir25a^{1}$ (BL41736), $Ir25a^{2}$ (BL41737), $Ir56b^{mimic}$ (BL27818), $ppk23^{G17320}$ (BL33300), $ppk23^{BG01654}$ (BL12571), $AKHR^{1}$, ZnT^{1} (BL31851), *Gr66a-Gal4* (BL57670), *elav-Gal4* (BL25750), *Ir25a-Gal4* (BL41728), *Ir76b-Gal4* (BL41730), *Ir56b-Gal4* (BL60706 and BL60707), *ppk23-Gal4*, *Gr64f-Gal4* (BL57669), *AKH-Gal4* (BL25684), *ppk23-LexA*, *AKHR-lexA*, *Ir76b-QF* (BL51312), *Gr66a-RFP* (BL60691), *UAS-tdTomato* (BL35837), *UAS-cd4-tdGFP*, *QUAS-GFP*, *LexAop-GFP*, *UAS-Dicer* (BL24650 and BL24651), *UAS-Kir2.1*, UAS-dTrpA1 (BL26263). And the following RNAi Lines were used: *Gr66a-1* (BL31284), *Gr66a-2* (BL31658), *Gr89a* (V8253), *Gr98b* (TH04882.N), *Ir76b* (BL54846), *Ir25a-1* (BL29539), *Ir25a-2* (BL43985), *Ir56b* (BL61274), *Ir56a* (BL60903), *Ir56d-1* (BL64617), *Ir56d-2* (TH04660N), *Ir75a* (BL67324), *Ir75c* (TH04833N), *Ir21a* (TH04627N), *Ir84a* (BL63992), *Ir48c* (BL45696), *Ir68b-1* (BL65360), *Ir68b-2* (TH05137N), *Ir68a* (BL60108), *Ir75b* (BL77143), *Ir8a* (BL25813), *Ir41a* (BL58056), *Ir11a* (BL61898), *Ir62a* (THU5140), *Ir75b* (BL77143), *Ir92a* (BL58205), *Ir94h* (BL53975), *Ir40a* (THU4000N), *Ir94b* (BL47552), *Ir94a* (BL53703), *Ir10a* (BL61842), *Ir76a* (BL34678), *AKH* (THU2417), *AKHR* (BL29577), *GFP* (TH00781.N).

Transgenic flies. The *Ir25a-LexA* line was generated by the homology assisted CRISPR knock-in (HACK) method as previously described (Lin and Potter, 2016; Zhang et al., 2020). Briefly, the donor vector pHACK-Gal4 > LexA was injected into embryos (*w-; Ir25a-Gal4/ nos-cas9;* +/+). We picked the files of successful injection by RFP signal in the compound eyes and then removed 3xP3-RFP by crossing them to an *Hsp70-Cre* line. The *UAS-Ir56b* line was generated by inserting the *Ir56b* coding sequence into the pJFRC81 vector and conventional P-element-mediated germ-line transformation. The *Ir56b* gene was cloned by amplifying two exon fragments from *Canton.S* genome by two pairs of primers, and the two fragments were ligated by fusion PCR.

primer1F: AAACGATTCATTCTAGATTAGTAATCGTAGCACTCAAATC;
primer1R: AGCAGGGCCTGGTACACCGGCAGCCAACGCAGCTCTT;
primer2F: TACCAGGCCCTGCT;
primer2R: AAACGATTCATTCTAGATTAGTAATCGTAGCACTCAAATC;
primer3F:
GGCGGCCGCGGCTCGAGGGTACCAACTTAAAAAAAAAAATCAAAATGCT
GCTTGACACGG ;
primer3R:
GCTCTAGATTAGTAATCGTAGCACTCAAATCGTTCATACTGTGGACG.

The files of successful transformation were picked up by the *mini-white* marker and then confirmed with PCR.

Two-way choice assay. The two-way choice assay was performed as previously described (Lee et al., 2018; Tanimura et al., 1982). Briefly, 50-80 3-5 days old flies were starved on an empty bottom with a small piece of Kim-wipe soaked with distilled water for 24h. The flies were then transferred to a 48-well plate which was loaded with 1% agarose with 5 mM sucrose or 5/15 mM sucrose plus salt solution with indicated concentration alternatively, and mixed with either blue (food blue NO.1, 0.125 mg/ml) or red food dye (food red NO.106 0.1 mg/ml). The flies were allowed to feed freely for 2h and frozen at -20 ℃. For calculating the preference index, the number of flies was counted with the color of their abdomens showing red (N_{red}) or blue (N_{blue}) or mixed purple (N_{purple}) under a stereomicroscope. The equation is:

$$preference\ index = \frac{(N_{red} + N_{purple}) - (N_{blue} + N_{purple})}{N_{red} + N_{blue} + N_{purple}}$$

Proboscis Extension Response assays. The proboscis extension response (PER) assays was performed as previously described (Shiraiwa and Carlson, 2007). Briefly, 3-5 days old flies were starved in an empty vial with a small piece of Kim-wipe soaked

with distilled water for 24 h. For the group of flies with zinc-deficiency diet, the vial contained 100 μM TPEN in 0.5% agarose. For group of normal food and external zinc supplement diet, we take vial with 5 mM zinc gluconate or indicated zinc salt in 0.5% Agarose. A female fly was gentle transferred into a 200 μL yellow pipette tip from an aspirator one by one and make sure the head and proboscis could move freely. 4% sucrose solution and double distilled water were given to the flies as positive and negative control before and after the test. Tastants were applied from low to high concentration and each tastant was tested 5 times to record the times of proboscis extension.

Immunohistochemistry. Immunostaining was carried our as previously described (Jaeger et al., 2018). Briefly, labella palps were dissected in PBS and fixed in 4% paraformaldehyde for 1h at 4 ℃. The samples were then washed with wash buffer (PBST, PBS containing 0.3% triton X-100) for 4x20 min and blocked in block buffer (0.5% goat serum in wash buffer) at 4 ℃ for 2 h. The samples were incubated in primary antibody at 4 ℃ overnight: rabbit anti-RFP (1:1000, 37249, Rockland) and chicken anti-GFP (1:1000, GFP-1020, AVES LABS). After washed with wash buffer for 4x20 min, the samples were incubated in second antibody at RT for 4 h: Alexa 488-goat anti-chicken (1:200, A11039, Invitrogen) and Alexa 555-goat anti-rabbit (1:200, A21428, Invitrogen). The samples were washed again with wash buffer for 6x20 min and mounted with wash buffer. All images were acquired using an Olympus FV1000 confocal microscope with a 20x air objective and processed with the *ImageJ* software.

RNA Extraction and RT-PCR

Total RNA was extracted using Trizol (Invitrogen), following the manufacturer's instructions. RNA extraction was conducted by using 50 mg flies of *Ir56b*^{mimic} or *Canton.S* in fresh weight and synthesized cDNA using AMP transcriptase (Promega).
The Ir56b cDNA was amplified using the primers:
Ir56b-primer F: GATCCGCTCGCCATATAGTT;
Ir56b-primer R: CGTCGATGGATAAAGAGCTC.
The cDNA of internal reference gene *tubulin* was amplified using the primers:
tubulin-primer F: TCCTTCTCGCGTGTGAAACA;
tubulin-primer R: CCGAACGAGTGGAAGATGAG.

Electrophysiology. Tip-recording was performed as previously described (Delventhal et al., 2014). Beadle-Ephrussi Ringer solution (B&E) as the reference electrode electrolyte (7.5 g NaCl, 0.35 g KCl, and 0.279 g $CaCl_2 \cdot 2H_2O$ in 1L ultrapure water, and 30 mM tricholine citrate solution (TCC) as the recording electrode electrolyte and

solvent for indicated tastants). 5-7 days old fly was anaesthetized on ice and the forelegs and midlegs were removed, for RNA interference for *AKHR*, the flies were maintained at 27℃ for 9 days before experiment. A reference electrode was inserted into the midline of the posterior dorsal thorax and was gently advanced anteriorly to the inside of the labellum until the proboscis is fully extended. L-type taste sensilla were test by 30 mM TCC, 104 mM sucrose, and S-type sensilla were test by 30 mM TCC, 100 mM BER before test by indicated tastants. The preparation was placed under an Olympus BW51I microscope equipped with a 32x air objective. The recording electrode was connected to a preamplifier (TastePROBE, Syntech, Hilversum, the Netherlands; http://www.ockenfels-syntech.com/), and the signals were collected and amplified by Digidata 1550B and Axoclamp 200B (Molecular Devices). Recordings of action potentials were acquired using a 10-kHz sampling rate. Spike traces were analyzed using the Clampfit software.

Manual feeding (MAFE) assay. To measure food ingestion, we performed the MAFE assay as previously described (Qi et al., 2015). Briefly, 3-5 days old flies were starved in vials with 100 μM TPEN in 0.5% agarose or vial with 5 mM zinc gluconate for 22 h at 4 ℃. Each fly was immobilized on a cover glass by nail polish after anaesthetized by CO_2. Flies were allowed to recovered for 2 h in a wet chamber with a small piece of Kim-wipe soaked with water at 25 ℃. A fine capillary (ASONE, 3-5998-02) with 1 μL 4% sucrose solution was advanced to the proboscis of the flies until they initiated ingestion. The length of liquid in capillary before and after feeding were measured by a vernier caliper and the volume of consumption was calculated.

Survival assays. Survival assay was performed as previously described (Lee et al., 2010; Lee et al., 2018). For the survival curve of *Canton.S*, 10 male and 10 female flies of 3 days old were transferred to a vial containing 100 mM fructose in 0.5% agarose plus zinc gluconate of indicated concentration. Viable flies were counted every 12 h and transferred to a new vial containing the same food for 8 days at 25 ℃. For survival test under the binary choice, 10 male and 10 female flies of days old were transferred to a plastic petri dish with four-quadrant dividers (diameter: 90 mm), which was loaded with 0.5% agarose with 100 mM fructose or 200 mM fructose plus 50 mM zinc gluconate, alternatingly. Viable flies were recorded every 12 h and transferred to a new dish containing the same food for 9 days at 25 ℃.

Quantification and statistical analysis. All error bars represent S.E.M.. Asterisks indicate significant differences from controls (*p < 0.05, **p < 0.01). All data statistical analysis was done using *GraphPad Prism 6* software. For two-way choice taste assay, we used one-way ANOVA followed by post hoc *t*-test with Bonferroni

correction. For PER assay, tip-recording electrophysiology and survival assay, we used one-way ANOVA or two-way ANOVA followed by post hoc *t*-test with Bonferroni correction or nonparametric Mann-Whitney test to analyze. For MAFE assay, we used nonparametric Mann-Whitney test. Each dot in bar plots represented indicated number of replications "n". For two-way choice taste assay "n" represented a group of tests with 50-80 files. For survival assay, the "n" represented the group number. In other assays, the "n" represented the number of individual flies.

REFERENCES

Delventhal, R., Kiely, A., and Carlson, J.R. (2014). Electrophysiological recording from Drosophila labellar taste sensilla. J Vis Exp, e51355.

Jaeger, A.H., Stanley, M., Weiss, Z.F., Musso, P.Y., Chan, R.C., Zhang, H., Feldman-Kiss, D., and Gordon, M.D. (2018). A complex peripheral code for salt taste in Drosophila. Elife *7*.

Lee, Y., Kim, S.H., and Montell, C. (2010). Avoiding DEET through insect gustatory receptors. Neuron *67*, 555-561.

Lee, Y., Poudel, S., Kim, Y., Thakur, D., and Montell, C. (2018). Calcium Taste Avoidance in Drosophila. Neuron *97*, 67-74 e64.

Lin, C.C., and Potter, C.J. (2016). Editing Transgenic DNA Components by Inducible Gene Replacement in Drosophila melanogaster. Genetics *203*, 1613-1628.

Qi, W., Yang, Z., Lin, Z., Park, J.Y., Suh, G.S., and Wang, L. (2015). A quantitative feeding assay in adult Drosophila reveals rapid modulation of food ingestion by its nutritional value. Mol Brain *8*, 87.

Rajan, A., Housden, B.E., Wirtz-Peitz, F., Holderbaum, L., and Perrimon, N. (2017). A Mechanism Coupling Systemic Energy Sensing to Adipokine Secretion. Dev Cell *43*, 83-98 e86.

Richards, C.D., Warr, C.G., and Burke, R. (2015). A role for dZIP89B in Drosophila dietary zinc uptake reveals additional complexity in the zinc absorption process. Int J Biochem Cell Biol *69*, 11-19.

Shiraiwa, T., and Carlson, J.R. (2007). Proboscis extension response (PER) assay in Drosophila. J Vis Exp, 193.

Tanimura, T., Isono, K., Takamura, T., and Shimada, I. (1982). Genetic Dimorphism in the Taste Sensitivity to Trehalose in Drosophila-Melanogaster. J Comp Physiol *147*, 433-437.

Zhang, L., Yu, J., Guo, X., Wei, J., Liu, T., and Zhang, W. (2020). Parallel Mechanosensory Pathways Direct Oviposition Decision-Making in Drosophila. Curr Biol *30*, 3075-3088 e3074.

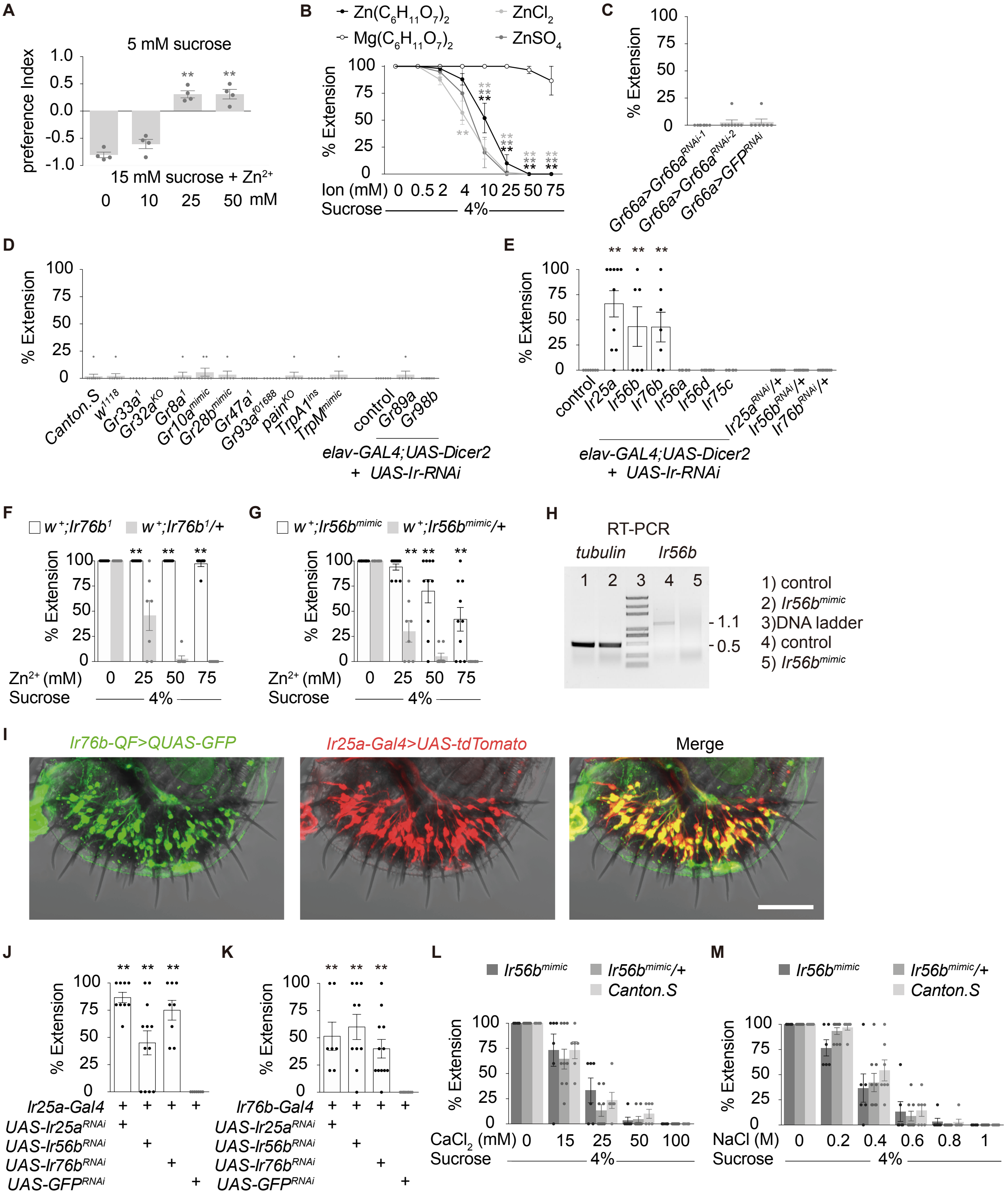
A
5 mM sucrose
preference Index
15 mM sucrose + Zn2+
0 10 25 50 mM
B
Zn(C6H11O7)2
Mg(C6H11O7)2
ZnCl2
ZnSO4
% Extension
Ion (mM) 0 0.5 2 4 10 25 50 75
Sucrose 4%
C
% Extension
Gr66a>Gr66aRNAi-1
Gr66a>Gr66aRNAi-2
Gr66a>GFPRNAi
D
% Extension
Canton.S
w1118
Gr33a1
Gr32aKO
Gr8a1
Gr10amimic
Gr28bmimic
Gr47a1
Gr93af01688
painKO
TrpA1ins
TrpMmimic
control
Gr89a
Gr98b
elav-GAL4;UAS-Dicer2
+ UAS-Ir-RNAi
E
% Extension
control
Ir25a
Ir56b
Ir76b
Ir56a
Ir56d
Ir75c
Ir25aRNAi/+
Ir56bRNAi/+
Ir76bRNAi/+
elav-GAL4;UAS-Dicer2
+ UAS-Ir-RNAi
F
w+;Ir76b1
w+;Ir76b1/+
% Extension
Zn2+ (mM) 0 25 50 75
Sucrose 4%
G
w+;Ir56bmimic
w+;Ir56bmimic/+
% Extension
Zn2+ (mM) 0 25 50 75
Sucrose 4%
H
RT-PCR
tubulin
Ir56b
1 2 3 4 5
1.1
0.5
1) control
2) Ir56bmimic
3)DNA ladder
4) control
5) Ir56bmimic
I
Ir76b-QF>QUAS-GFP
Ir25a-Gal4>UAS-tdTomato
Merge
J
% Extension
Ir25a-Gal4
UAS-Ir25aRNAi
UAS-Ir56bRNAi
UAS-Ir76bRNAi
UAS-GFPRNAi
K
% Extension
Ir76b-Gal4
UAS-Ir25aRNAi
UAS-Ir56bRNAi
UAS-Ir76bRNAi
UAS-GFPRNAi
L
Ir56bmimic
Ir56bmimic/+
Canton.S
% Extension
CaCl2 (mM) 0 15 25 50 100
Sucrose 4%
M
Ir56bmimic
Ir56bmimic/+
Canton.S
% Extension
NaCl (M) 0 0.2 0.4 0.6 0.8 1
Sucrose 4%

Figure S1

Figure S1. Zinc taste is independent of bitter receptors. (A) Preference of files to 5 mM sucrose alone versus 15 mM sucrose with zinc gluconate of indicated concentrations. zinc gluconate reversed flies' preference to less sweet sucrose solution. One-way ANOVA followed by post hoc t test with Bonferroni correction; n = 4. (B) Fraction of WT flies showing PER to different concentrations of zinc or magnesium salt supplied in 4% sucrose, which demonstrates dose-dependent aversion for Zn^{2+} but not Mg^{2+}. Two-way ANOVA followed by post hoc t test with Bonferroni correction; n = 6 to 13. (C) PER of files with genetic knockdown of Gr66a to 25 mM zinc gluconate in 4%. One-way ANOVA followed by post hoc t test with Bonferroni correction; n = 7 to 8. (D) PER of files with mutant or genetic knockdown of aversive taste chemoreceptors to 4% sucrose containing 50 mM zinc gluconate indicates these receptors was dispensable for zinc detection. One-way ANOVA followed by post hoc t test with Bonferroni correction; n = 6 to 12. (E) PER of files with genetic knockdown of candidate IRs to 50 mM zinc gluconate in 4% sucrose. One-way ANOVA followed by post hoc t test with Bonferroni correction; n = 6 to 10. (F and G) Dose-dependent taste aversion of *Ir76b, Ir56b* mutants which were backcrossed to *Canton.S* to zinc gluconate. Two-way ANOVA followed by post hoc t test with Bonferroni correction; n = 7 to 10. (H) Confirmation of $Ir56b^{mimic}$ as a mutant by RT-PCR. The expression of Ir56b in *Canton.S* was used as control. *tubulin* was used as the reference gene. The sizes of the RT-PCR products are indicated in kb. (I) Expression of *Ir76b-QF* (*GFP*) and *Ir25a-Gal4* (*tdTomato*) on the labellum. (Scale bars: 50 μm.) (J and K) PER of files with genetic knockdown of Ir25a, r56b or Ir76b to 50mM zinc gluconate supplement of 4% sucrose in Ir25a neurons (J) and in Ir76b neurons (K), respectively. One-way ANOVA followed by post hoc t test with Bonferroni correction; n = 7 to 12. (L and M) Ir56b mutant showed normal PER to indicated concentration of Ca^{2+} and Na^{+}. Two-way ANOVA followed by post hoc t test with Bonferroni correction; n = 6 to 7. For all comparisons performed in this figure, *$p < 0.05$, **$p < 0.01$.

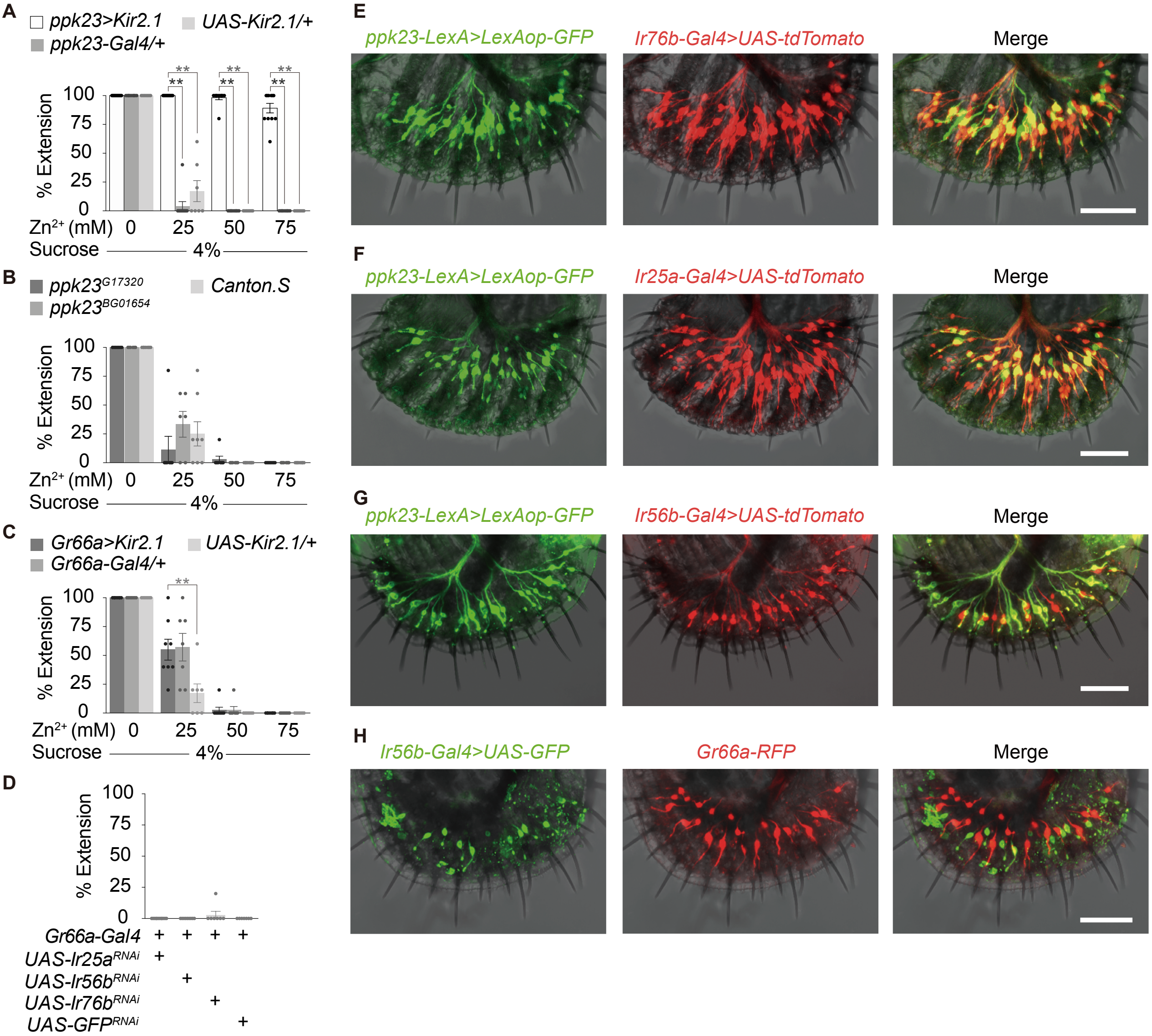
A
ppk23>Kir2.1
UAS-Kir2.1/+
ppk23-Gal4/+
% Extension
100
75
50
25
0
**
Zn2+ (mM) 0 25 50 75
Sucrose 4%
B
ppk23G17320
Canton.S
ppk23BG01654
% Extension
100
75
50
25
0
Zn2+ (mM) 0 25 50 75
Sucrose 4%
C
Gr66a>Kir2.1
UAS-Kir2.1/+
Gr66a-Gal4/+
% Extension
100
75
50
25
0
**
Zn2+ (mM) 0 25 50 75
Sucrose 4%
D
% Extension
100
75
50
25
0
Gr66a-Gal4 + + + +
UAS-Ir25aRNAi +
UAS-Ir56bRNAi +
UAS-Ir76bRNAi +
UAS-GFPRNAi +
E
ppk23-LexA>LexAop-GFP
Ir76b-Gal4>UAS-tdTomato
Merge
F
ppk23-LexA>LexAop-GFP
Ir25a-Gal4>UAS-tdTomato
Merge
G
ppk23-LexA>LexAop-GFP
Ir56b-Gal4>UAS-tdTomato
Merge
H
Ir56b-Gal4>UAS-GFP
Gr66a-RFP
Merge

Figure S2

Figure S2. Zinc taste is mediate with a subset of ppk23 salt GRNs. (A) PER of files with inhibition of ppk23 GRNs activity to zinc gluconate supplement of 4% sucrose shows dull zinc taste. Two-way ANOVA followed by post hoc t test with Bonferroni correction; n = 7 to 11. (B) ppk23 mutant's PER to zinc gluconate. Two-way ANOVA followed by post hoc t test with Bonferroni correction; n = 6 to 8. (C) Files with inhibition of bitter GRNs activity shows similar PER to WT towards zinc gluconate. Two-way ANOVA followed by post hoc t test with Bonferroni correction; n = 7 to 8. (D) PER of files with genetic knockdown of Ir25a, r56b or Ir76b in bitter GRNs to 50mM zinc gluconate supplement of 4% sucrose. One-way ANOVA followed by post hoc t test with Bonferroni correction; n = 7 to 14. (E) Expression of *ppk23-LexA* (*GFP*) and *Ir76b-Gal4* (*tdTomato*) on the labellum. (Scale bars: 50 μm.) (F) Expression of *ppk23-LexA* (*GFP*) and *Ir25a-Gal4* (*tdTomato*) on the labellum. (Scale bars: 50 μm.) (G) Expression of *ppk23-LexA* (*GFP*) and *Ir56b-Gal4* (*tdTomato*) on the labellum. (Scale bars: 50 μm.) (H) Expression of *Ir56b-Gal4* (*GFP*) and *Gr66a-RFP* on the labellum. (Scale bars: 50 μm.) For all comparisons performed in this figure, $*p < 0.05$, $**p < 0.01$.

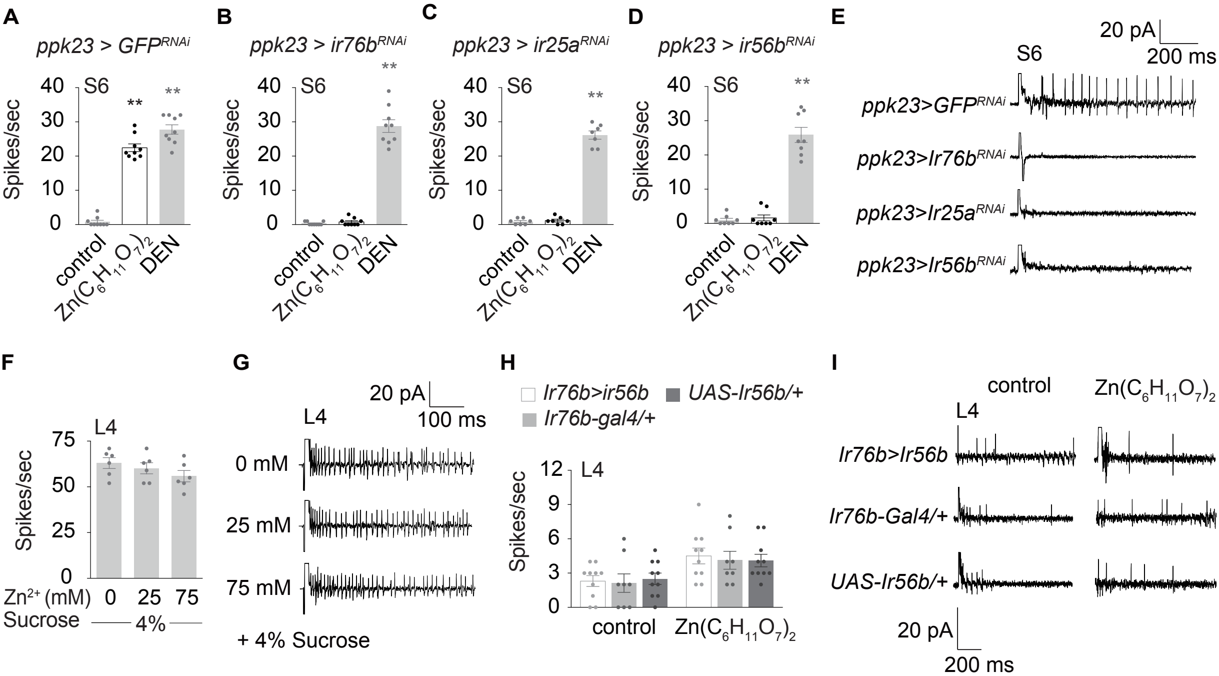

A
ppk23 > GFP^RNAi
S6
Spikes/sec
40
30
20
10
0
**
**
control
Zn(C6H11O7)2
DEN
B
ppk23 > ir76b^RNAi
C
ppk23 > ir25a^RNAi
D
ppk23 > ir56b^RNAi
E
20 pA
200 ms
ppk23>GFP^RNAi
ppk23>Ir76b^RNAi
ppk23>Ir25a^RNAi
ppk23>Ir56b^RNAi
F
L4
75
50
25
0
Zn2+ (mM) 0 25 75
Sucrose 4%
G
20 pA
100 ms
0 mM
25 mM
75 mM
+ 4% Sucrose
H
Ir76b>ir56b
UAS-Ir56b/+
Ir76b-gal4/+
12
9
6
3
0
control
Zn(C6H11O7)2
I
control
Zn(C6H11O7)2
Ir76b>Ir56b
Ir76b-Gal4/+
UAS-Ir56b/+
20 pA
200 ms

Figure S3

Figure S3. Zinc did not inhibit the activity of sugar GRNs.
(A to D) Genetic knockdown of Ir76b, Ir25a or Ir56b in taste neurons diminished the zinc-triggered spikes to zinc gluconate but not DEN compared with control. One-way ANOVA followed by post hoc t test with Bonferroni correction; n = 7 to 9. (E) Representative traces of spikes from indicated genotype of flies to 75mM zinc gluconate. (F) Tip recordings from L4 sensilla shows zinc did not suppress sugar induced action potentials. One-way ANOVA followed by post hoc t test with Bonferroni correction; n = 6. (G) The representative traces of spikes from L4 sensilla in response to indicated concentrations of zinc gluconate in 4% sucrose. (H) Tip recordings from L4 sensilla of flies with ectopic expression of Ir56b in Ir76b expressing neurons. Two-way ANOVA followed by post hoc t test with Bonferroni correction; n = 8 to 10. (I) The representative traces of spikes from L4 sensilla of indicated genotype of flies to 75 mM zinc gluconate or control. For all comparisons performed in this figure, *$p < 0.05$, **$p < 0.01$.

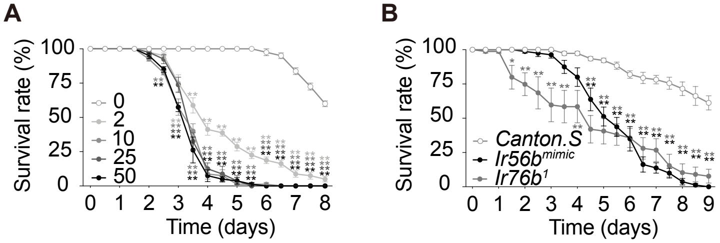
A
Survival rate (%)
100
75
50
25
0
0
2
10
25
50
0 1 2 3 4 5 6 7 8
Time (days)
B
Survival rate (%)
100
75
50
25
0
Canton.S
Ir56b^mimic
Ir76b^1
0 1 2 3 4 5 6 7 8 9
Time (days)

Figure S4

Figure S4. Zinc avoidance is critical for flies' survival. (A) Survival of *Canton.S* flies fed 100 mM fructose mixed with indicated concentration of zinc gluconate solution in agarose. Two-way ANOVA followed by post hoc t test with Bonferroni correction; n = 4.

(B) Survival of Ir56b and Ir76b mutants fed 100 mM fructose or 200 mM fructose mixed with indicated concentration of zinc gluconate solution in agarose. Two-way ANOVA followed by post hoc t test with Bonferroni correction; n = 4.

For all comparisons performed in this figure, *p < 0.05, **p < 0.01.

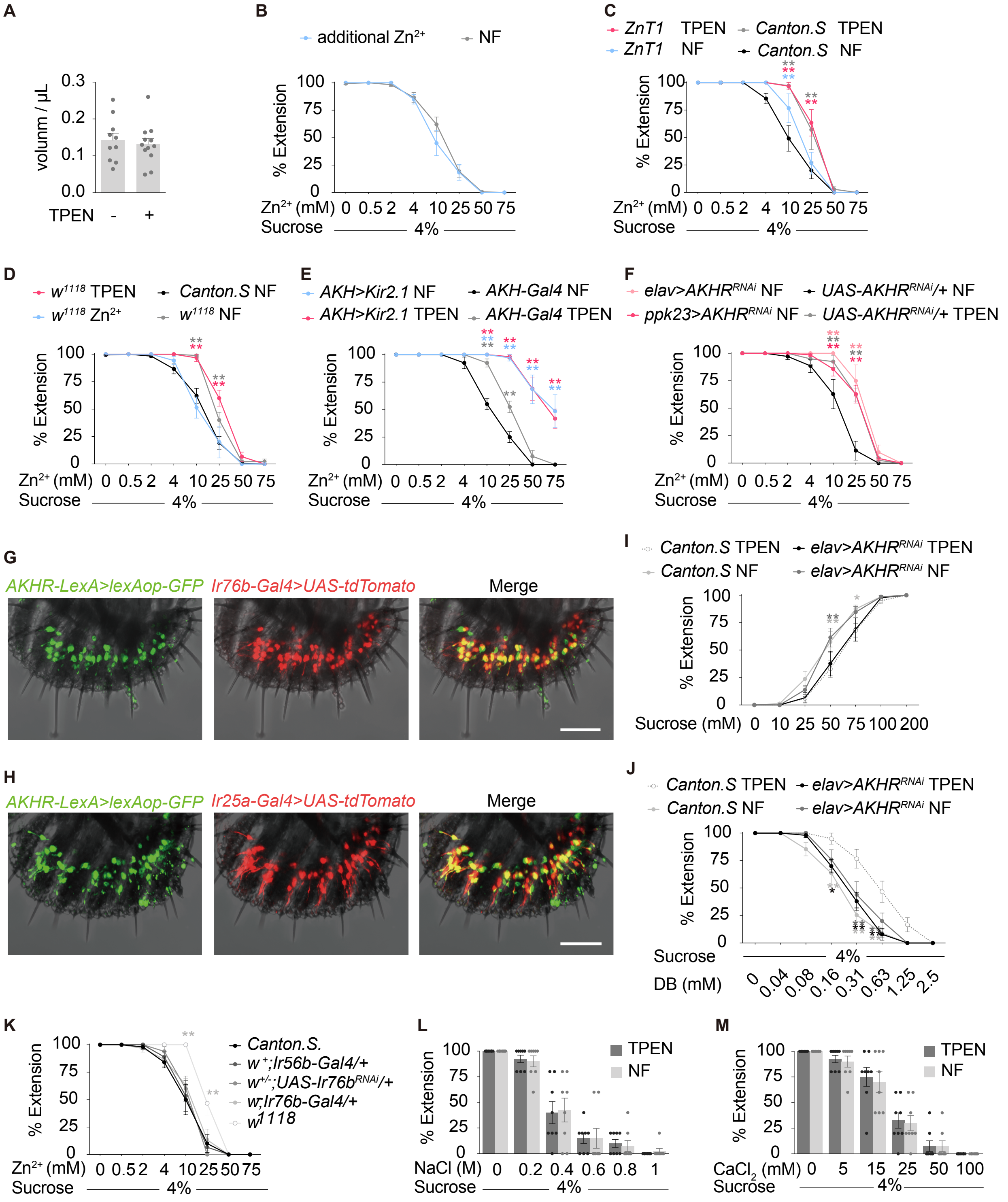

A
volumn / μL
TPEN - +
B
additional Zn2+
NF
% Extension
Zn2+ (mM) 0 0.5 2 4 10 25 50 75
Sucrose 4%
C
ZnT1 TPEN
ZnT1 NF
Canton.S TPEN
Canton.S NF
D
w1118 TPEN
w1118 Zn2+
Canton.S NF
w1118 NF
E
AKH>Kir2.1 NF
AKH>Kir2.1 TPEN
AKH-Gal4 NF
AKH-Gal4 TPEN
F
elav>AKHRRNAi NF
ppk23>AKHRRNAi NF
UAS-AKHRRNAi/+ NF
UAS-AKHRRNAi/+ TPEN
G
AKHR-LexA>lexAop-GFP
Ir76b-Gal4>UAS-tdTomato
Merge
H
AKHR-LexA>lexAop-GFP
Ir25a-Gal4>UAS-tdTomato
Merge
I
Canton.S TPEN
Canton.S NF
elav>AKHRRNAi TPEN
elav>AKHRRNAi NF
Sucrose (mM) 0 10 25 50 75 100 200
J
Canton.S TPEN
Canton.S NF
elav>AKHRRNAi TPEN
elav>AKHRRNAi NF
Sucrose 4%
DB (mM) 0 0.04 0.08 0.16 0.31 0.63 1.25 2.5
K
Canton.S.
w+;Ir56b-Gal4/+
w+/-;UAS-Ir76bRNAi/+
w;Ir76b-Gal4/+
w1118
L
TPEN
NF
NaCl (M) 0 0.2 0.4 0.6 0.8 1
Sucrose 4%
M
TPEN
NF
CaCl2 (mM) 0 5 15 25 50 100
Sucrose 4%

Figure S5

Figure S5. AKH-AKHR axis is required for zinc taste regulation. (A) Sucrose intake of files reared on zinc-depleted or normal food diet. Nonparametric Mann-Whitney test; n = 10 to 12. (B) PER of WT flies with additional zinc supplement to the food or normal food diet to different concentration of zinc gluconate supplement of 4% sucrose. Two-way ANOVA followed by post hoc t test with Bonferroni correction; n = 12. (C) PER of WT flies and *ZnT1* mutant with zinc-depleted or normal food diet to zinc gluconate supplement of 4% sucrose. Two-way ANOVA followed by post hoc t test with Bonferroni correction; n = 6 to 11. (D) PER of WT and w^{1118} flies with zinc-deficient, normal food or additional zinc supplement diet shows taste sensitivity to zinc of w^{1118} flies. Two-way ANOVA followed by post hoc t test with Bonferroni correction; n = 6 to 12. (E) PER of flies with inhibition of AKH producing neurons on zinc-deficient or normal food diet. Two-way ANOVA followed by post hoc t test with Bonferroni correction; n = 7 to 11. (F) PER of flies with genetic knockdown of AKHR pan-neuronally or in ppk23 neurons. Two-way ANOVA followed by post hoc t test with Bonferroni correction; n = 7 to 14. (G) Expression of *AKHR-LexA* (*GFP*) and *Ir76b-Gal4* (*tdTomato*) on the labellum. (Scale bars: 50 µm.) (H) Expression of *AKHR-LexA* (*GFP*) and *Ir25a-Gal4* (*tdTomato*) on the labellum. (Scale bars: 50 µm.) (I and J) PER of WT and genetic knockdown of AKHR pan-neuronally flies with zinc-deficient or normal food diet to sucrose, or denatonium benzoate. Two-way ANOVA followed by post hoc t test with Bonferroni correction; n = 9 to 20.
(K) PER of flies with different copies of *white* (*mini-white*) transgenes. Two-way ANOVA followed by post hoc t test with Bonferroni correction; n = 6 to 10. (L and M) ppk23>GFP^{RNAi} files on zinc-deficiency diet or normal diet shows comparable sensitivity to Ca^{2+} or Na^{+}. Two-way ANOVA followed by post hoc t test with Bonferroni correction; n = 8. For all comparisons performed in this figure, *$p < 0.05$, **$p < 0.01$.
